# ADAMTS3 Promotes Hepatocellular Carcinoma Cell Growth and Migration Through TGF-β:TGFBR2 Signaling

**DOI:** 10.3390/cells15191784

**Published:** 2026-09-30

**Authors:** Chen Wang, Huaizhou Jin, Qijie Zhang, Yanqiu Zou, Yaosen Wang, Nicolò Maccaferri, Ali Douaki, Jianguo Feng, Denis Garoli, Shangzhong Jin

**Affiliations:** 1 College of Optical and Electronic Technology, China Jiliang University, Hangzhou 310018, China; 2 Key Laboratory of Quantum Precision Measurement, College of Physics, Zhejiang University of Technology, Hangzhou 310018, China; 3 Second Affiliated Hospital, School of Medicine, Zhejiang University, Hangzhou 310018, China; 4 College of Optical Science and Engineering, Zhejiang University, Hangzhou 310027, China; 5 Ultrafast Nanoscience Group, Department of Physics, Umeå University, Linnaeus väg 24, 901 87 Umeå, Sweden; 6 Optoelectronic Department, Istituto Italiano di Tecnologia, Via Morego 30, 16163 Genova, Italy; 7 Dipartimento di Scienze e Metodi dell’Ingegneria, Università degli Studi di Modena e Reggio Emilia, Viale Amendola 2, 14122 Reggio Emilia, Italy; 8 Experimental Research Center, Zhejiang Cancer Hospital, Hangzhou 310022, China; 9 Umeå Centre for Microbial Research, Umeå University, Linnaeus väg 24, 901 87 Umeå, Sweden; 10 Wallenberg Initiative Materials Science for Sustainability, Department of Physics, Umeå University, Linnaeus väg 24, 901 87 Umeå, Sweden

**Keywords:** ADAMTS3, hepatocellular carcinoma, DNA origami, SERS

## Abstract

Early diagnosis of hepatocellular carcinoma (HCC) still represents a significant challenge, particularly in the context of etiological shift toward obesity and non-viral liver disease-associated HCC. Through intersection-based analysis of independent transcriptomic datasets comprising healthy controls, individuals with obesity or NAFLD, and HCC patients with viral or non-viral etiologies, we prioritized ADAMTS3, a secreted protein, for further investigation of its functional and mechanistic relevance to HCC. ADAMTS3 was upregulated in HCC and demonstrated transcriptomic discrimination between HCC and non-HCC populations. Notably, in the validation cohort, ADAMTS3 retained discriminative capacity in clinically defined alpha-fetoprotein-negative HCC patients. Functionally, ADAMTS3 knockdown impaired cell proliferation, clonogenicity, and migration, while inducing apoptosis and cell-cycle arrest. Reintroduction of ADAMTS3 partially rescued these cellular effects. Mechanistically, ADAMTS3 promotes HCC cell growth and migration through TGF-β:TGFBR2 signaling. ADAMTS3 knockdown increased extracellular TGF-β and downstream SMAD phosphorylation. Neutralization of extracellular TGF-β alleviated growth inhibition caused by ADAMTS3 knockdown. Consistently, TGFBR2 knockdown partially restored the cell growth and migration suppressed by ADAMTS3 knockdown. We also developed a DNA origami-based SERS aptasensor targeting ADAMTS3, achieving highly sensitive detection of ADAMTS3 down to 10^−12^ M with strong quantitative capability (R^2^ = 0.914). Together, our findings support a role for ADAMTS3 in promoting HCC cell growth and migration and provide a basis for further investigation of its relevance to HCC biomarker development. The DNA origami-based SERS aptasensor provides a sensitive analytical approach for future studies of circulating or serum ADAMTS3.

## 1. Introduction

Liver cancer ranks as the third leading cause of cancer-related deaths worldwide, with hepatocellular carcinoma (HCC) representing about 80% of cases [1]. Unlike most other major cancers, mortality rates for HCC have shown limited improvement over the past decade [2]. For patients with early-stage disease (Barcelona Clinic Liver Cancer stage A), surgical options remain the treatment of choice, offering curative potential: median overall survival (OS) exceeds 5 years following resection and can surpass 10 years after liver transplantation [3,4]. Unfortunately, nearly 70% of patients are diagnosed at an advanced stage, when curative therapies are no longer viable [2,5]. This highlights the urgent global need for more effective strategies enabling earlier detection of HCC. And this is particularly pressing given the epidemiological transition in HCC burden from virus-related disease to causes such as alcohol-associated liver injury and obesity, conditions in which current screening tools are less effective [6,7,8,9,10,11].

To explore secreted proteins relevant to the evolving landscape of HCC etiology, we performed a transcriptome-based screening across four independent sample groups. This analysis revealed candidates including ADAMTS3. Notably, ADAMTS3 also acts as a mechanistic node that promotes HCC cell growth and migration. Although relatively underexplored, ADAMTS3 is localized at or near the cell surface, where it functions as the principal N-propeptidase of procollagen II to initiate collagen fibril assembly, a key structural component of the extracellular matrix (ECM), and as a VEGF-C-activating protease that regulates lymphangiogenesis and angiogenesis [12,13]. In cancer biology, limited studies suggest that ADAMTS3 plays diverse roles, acting as a tumor promoter in glioma stem cells but exhibiting tumor-suppressive effects in breast cancer [14]. Its function in HCC, however, remains largely unclear and has not been systematically explored. In this study, we demonstrate that ADAMTS3 maintains malignant properties in HCC cells through the TGF-β:TGFBR2 axis.

Sensitive analytical approaches for ADAMTS3 detection may facilitate future evaluation in clinical cohorts. Surface-Enhanced Raman Scattering (SERS) represents a powerful tool for detection proteins, offering ultrahigh sensitivity, reaching femtomolar or even single-molecule levels, together with molecular fingerprint-level specificity [15]. Owing to these features, SERS has been widely explored for biomarker-based in cancer research, benefiting from its exceptional sensitivity and noninvasive nature [16]. However, its practical application is frequently limited by the need for stable electromagnetic “hotspots,” which are the dominant contributors to SERS signal enhancement and are critical for ensuring reliable detection [17]. DNA origami technology, first introduced by Paul Rothemund in 2006, has emerged as a versatile platform for the programmable folding of DNA into defined shapes and patterns [18]. This enables the construction of nanoscale architectures with precise spatial control, allowing the organized arrangement of plasmonic nanoparticles to produce reproducible plasmonic “hotspots.” In addition, DNA origami structures are highly adaptable to chemical modification, making them particularly suitable for analytical applications [19]. Therefore, we further developed a DNA origami-based aptasensor capable of sensitive and selective detection of ADAMTS3.

Collectively, based on the shifting landscape of HCC etiology, we prioritized ADAMTS3 for further investigation through intersection-based analysis of high-throughput transcriptomic datasets from healthy individuals and patients with obesity, non-alcoholic fatty liver disease (NAFLD), virus-associated HCC, and both early- and late-stage HCC. We then investigated the functional and mechanistic relevance of ADAMTS3 to HCC in vitro and developed a DNA origami-based aptasensor for the sensitive and selective detection of ADAMTS3, providing a platform for future studies on ADAMTS3.

## 2. Materials and Methods

### 2.1. Differential Expression Analysis

The data used in this study are publicly available (GSE164359, GSE141200, GSE126848, GSE199819, PRJNA912860, PRJNA715360, PRJNA743855). All corresponding analyses were performed using R (version 4.4.1). The differential expressed genes (DEGs) were identified through the application of the DESeq2 package, with the cutoff criteria (adjusted *p*-value < 0.05 and |log_2_FoldChange| > 1). ComBat was applied to reduce dataset-associated technical variation. The diagnostic performance of candidate genes was assessed using the pROC package. Candidates were subjected to LASSO (least absolute shrinkage and selection operator) analysis, and the LASSO regression model was implemented with the glmnet package. Identified DEGs were subsequently analyzed for Gene Ontology (GO) terms and Kyoto Encyclopedia of Genes and Genomes (KEGG) pathway enrichment using the clusterProfiler package, and the results were visualized with the ggplot2 package. A significance threshold of *p* < 0.05 was applied throughout the analyses.

### 2.2. Cell Lines

The Huh7 and HEK293T cells were obtained from Immocell (Xiamen, China) and American Type Culture Collection (ATCC, Manassas, VA, USA), respectively. Hep3B cells were kindly provided by Prof. Gu at Zhejiang University and maintained in MEM (Yuanye Bio-technology, Shanghai, China) supplemented with 10% FBS (Thermo Fisher, Waltham, MA, USA), 1% non-essential amino acids (Yuanye Bio-technology, Shanghai, China), and penicillin/streptomycin (Beyotime, Shanghai, China) [20]. HEK293T and Huh7 cells were maintained in DMEM (Yuanye Bio-technology, Shanghai, China) media with 10% FBS and penicillin/streptomycin. All cells were maintained at 37 °C in a humidified atmosphere (Thermo Fisher, Waltham, MA, USA) with 5% CO_2_ and all the experiments were performed with cell lines cultured for <4 months.

### 2.3. Preparation of Expression Vectors

Expression plasmids were generated using a Hieff Clone^®^ Universal II One Step Cloning Kit (Yeasen Biotechnology, Shanghai, China), according to the manufacturer’s protocol. Primers used to construct both wild-type or shRNA-resistant ADAMTS3/TGFBR2 are listed in Supplementary Table S1. Backbone vectors were digested with NheI and NotI restriction endonucleases (New England Biolabs, Ipswich, MA, USA). *E. coli* DH5a (Yeasen Biotechnology, Shanghai, China) was used for plasmid cloning. shRNAs targeted for ADAMTS3/TGFBR2 were cloned into AgeI and EcoRI digested piggybac-U6 vector with NEB Quick Ligation™ Kit (New England Biolabs, Ipswich, MA, USA). The corresponding sequences for the shRNAs are listed in Table S1.

### 2.4. Proliferation Assay

Cell Counting Kit-8 (CCK-8, APExBIO, Boston, MA, USA) and direct counting were used to perform proliferation assays. For CCK-8-based proliferation, 1.5 × 10^3^ cells were plated in 100 µL normal growth medium in 96-well plates and 10 µL of the CCK-8 solution was added to each well. The absorbance at 450 nm was measured using a microplate reader (Multiskan FC, Thermo Fisher, Waltham, MA, USA) after incubation for 1 h. For cell counting-based proliferation, cells were seeded in a 6-well plate with a concentration of 2 × 10^4^ cells per well. Cell numbers were determined by counting trypan blue-negative cells using a hemocytometer under microscopy. Cells were counted every 24 h after seeding.

### 2.5. Colony Formation Assay

For each group, 800 cells were seeded in six-well plates in 2 mL of medium. Fresh medium was added after 96 h to prevent nutrient exhaustion. At the endpoint, colonies were fixed with 4% Paraformaldehyde Fix Solution (P0099, Beyotime, Shanghai, China) for 10 min at room temperature, then washed twice with PBS. The samples were subsequently stained with crystal violet solution (C0121, Beyotime, Shanghai, China) at 37 °C for 10 min and rinsed thoroughly with water. ImageJ (v1.54) was used for the quantification of area covered by colonies and colony number.

### 2.6. Western Blot Analysis

A total of 5 × 10^5^ cells/well were seeded into 6-well plates and incubated for 48 h. Cell samples were rinsed thoroughly with PBS. Total cellular protein was extracted on ice using Mammalian Protein Extraction Reagent (M-PER™, Thermo Scientific, Waltham, MA, USA) supplemented with Halt™ Protease Inhibitor Cocktail (Thermo Fisher, Waltham, MA, USA). Supernatants were then collected by centrifugation at 18,000× *g* for 10 min at 4 °C. Cell extracts were diluted with loading buffer to a final protein concentration of 1 mg/mL, separated by SDS-PAGE, and transferred onto polyvinylidene difluoride (PVDF) membranes (Bio-Rad, Hercules, CA, USA). The membranes were then blocked with 5% non-fat milk. After three washes with TBST, membranes were reacted with a target antibody overnight at 4 °C. The membranes were further washed with TBST and incubated with a horseradish peroxidase-conjugated secondary antibody for 1 h at room temperature. After another wash step, the membranes were reacted with a BeyoECL Plus working solution (P0018S, Beyotime, Shanghai, China).

### 2.7. DNA Origami Assembly

DNA staple strands (0.1 µM each) were pooled by combining 0.5 µL from each tube into a total volume of 101 µL (sequences are listed in Supplementary Table S1). The pooled mixture was vortexed thoroughly and diluted to 1% by mixing 1 µL of the pooled staples with 99 µL of ultrapure water. To assemble DNA origami, an annealing solution (100 µL total) was prepared by combining 20 µL of the diluted staple mixture, 10 µL of 10× TAE buffer supplemented with 0.1 M MgCl_2_, 67.5 µL of ultrapure water, and 2.5 µL of m13mp18 scaffold (250 µg/mL). The solution was subjected to a thermal annealing protocol, which consisted of heating to 80 °C followed by gradual cooling to 20 °C. Assembled DNA origami structures were purified by agarose gel electrophoresis (1% agarose in 1× TAE containing 0.1 M MgCl_2_) at 75 V for 60 min. The desired DNA band was excised and extracted using the SanPrep Column DNA Gel Extraction Kit (Sangon, Shanghai, China), yielding 25 µL of purified DNA origami.

### 2.8. DNA Origami-Gold Nanoparticle Conjugation

DNA origami-gold nanoparticle constructs were fabricated according to the method reported by Tapio et al. [21]: 25 µL of purified DNA origami was incubated with 2 µL of TCEP to reduce and activate thiol groups. In parallel, 100 µL of gold nanoparticle solution was centrifuged at 2900× *g* for 5 min. The supernatant was discarded, and the pellet was resuspended in 26.5 µL of ultrapure water containing 3.5 µL of 0.2% SDS. The activated DNA origami was then mixed with the prepared gold nanoparticles and incubated at 40 °C for 40 min without agitation. To facilitate salt-aging, NaBr was sequentially added at 10 min intervals with gentle shaking in the following volumes and concentrations: 1.7 µL (0.4 M), 2.1 µL (0.4 M), 2.3 µL (0.4 M), 2.3 µL (1 M), 2.3 µL (1 M), 2.3 µL (1 M), and 6.5 µL (1 M). This was followed by the addition of 6 µL and 6.6 µL of 1× TAE buffer containing 5 mM MgCl_2_ at 10 min intervals. The conjugated product was purified through four washing steps, each involving centrifugation at 2900× *g* for 5 min, removal of the supernatant, and resuspension in 20 µL of fresh buffer. The first two washes were performed using 1× TAE with 5 mM MgCl_2_ and 0.02% SDS, while the final two washes used 1× TAE with 5 mM MgCl_2_ without SDS.

### 2.9. Raman Spectroscopy Detection

DNA origami-gold nanoparticles were incubated with either 1X PBS (blank) or purified Histagged-ADAMTS3 at diverse concentrations. A 10 µL aliquot of each mixture was deposited onto a silicon wafer and subjected to Raman spectroscopy. Measurements were performed on a Horiba XploRA Plus Raman microscope (Horiba, Kyoto, Japan) equipped with a 50× long-focal-length objective (NA = 0.75). Visible Raman spectra were recorded using a 633 nm excitation laser set at 3.5% power.

### 2.10. Synthesis of AuNPs

AuNPs were synthesized using a modified Turkevich–Frens method, as detailed by Smirnov [22]. In brief, an aqueous solution of hydrogen tetrachloroaurate (III) was heated to boiling under continuous stirring, and a reducing agent, ascorbic acid, was added dropwise. This reduction led to the formation of colloidal gold, with particle size and distribution controlled by the type and concentration of the reducing agent. The resulting nanoparticles were stable in colloidal suspension and suitable for subsequent biosenor construction.

### 2.11. In Silico Aptamer Screening

The three-dimensional structure of the target protein was obtained from AlphaFold 3 and used as input for the Aptaloop screening pipeline [23]. The predicted binding pocket was identified using the pocket detection algorithm CASTpFold prior to aptamer generation [24]. The top three candidates were subsequently subjected to three-dimensional structure modeling using FARFAR2 [25], followed by molecular docking with the target protein using HADDOCK [26]. All computational analyses were performed using default parameters unless otherwise specified.

### 2.12. SPRi Assay

The chip was prepared according to the method in our previous work [27]. Briefly, the separated bare Au chips were washed with fresh piranha solution (H_2_SO_4_/H_2_O_2_, 7:3) for 30 min at 37 °C, then cleaned with ultrapure water and dried. A 0.8 μL sample of 5 μM thiol-modified aptamers (his-tag aptamer as negative control) was deposited on the gold chip by a homemade spotter. Proteins (20 μg/mL) were immobilized through EDC/NHS-mediated amine coupling on a carboxyl-functionalized SAM-modified gold surface. Chips were incubated overnight at 80% relative humidity, then cleaned and blocked for 30 min with 1 mM MCH solution. SPRi testing was conducted following the cleaning of the loading and injection lines.

### 2.13. Wound-Healing Assay

A total of 4 × 10^5^ cells/well were seeded in a 6-well plate. After cultivation in a cell incubator with 5% CO_2_ at 37 °C for 8 h, the cells were incubated with a serum-free medium for 24 h. A wound was generated in the adherent cell monolayer using a standard 200 µL pipette tip and a ruler. Then, the cells were continually cultivated in the serum-free medium for 12 h. Images were captured using an inverted light microscope (Olympus, Tokyo, Japan).

### 2.14. Cell-Cycle Distribution Assay

A total of 5  ×  10^5^ cells per sample were seeded into 6-well plates and incubated for 48 h. Cells were harvested, washed with PBS, and fixed in 70% ethanol overnight at −20 °C. Fixed cells were washed with PBS to remove residual ethanol. Cells were stained with propidium iodide (PI)/Tirton X-100 staining solution in the presence of RNase A (Sigma, Merck, St. Louis, MO, USA) for 3 h at room temperature. Cells were sampled for FACS analysis (FACScan, BD, Franklin Lakes, NJ, USA).

## 3. Results and Discussion

### 3.1. Identification of ADAMTS3 Through Intersection-Based Transcriptomic Analysis in HCC

**Figure 1:**
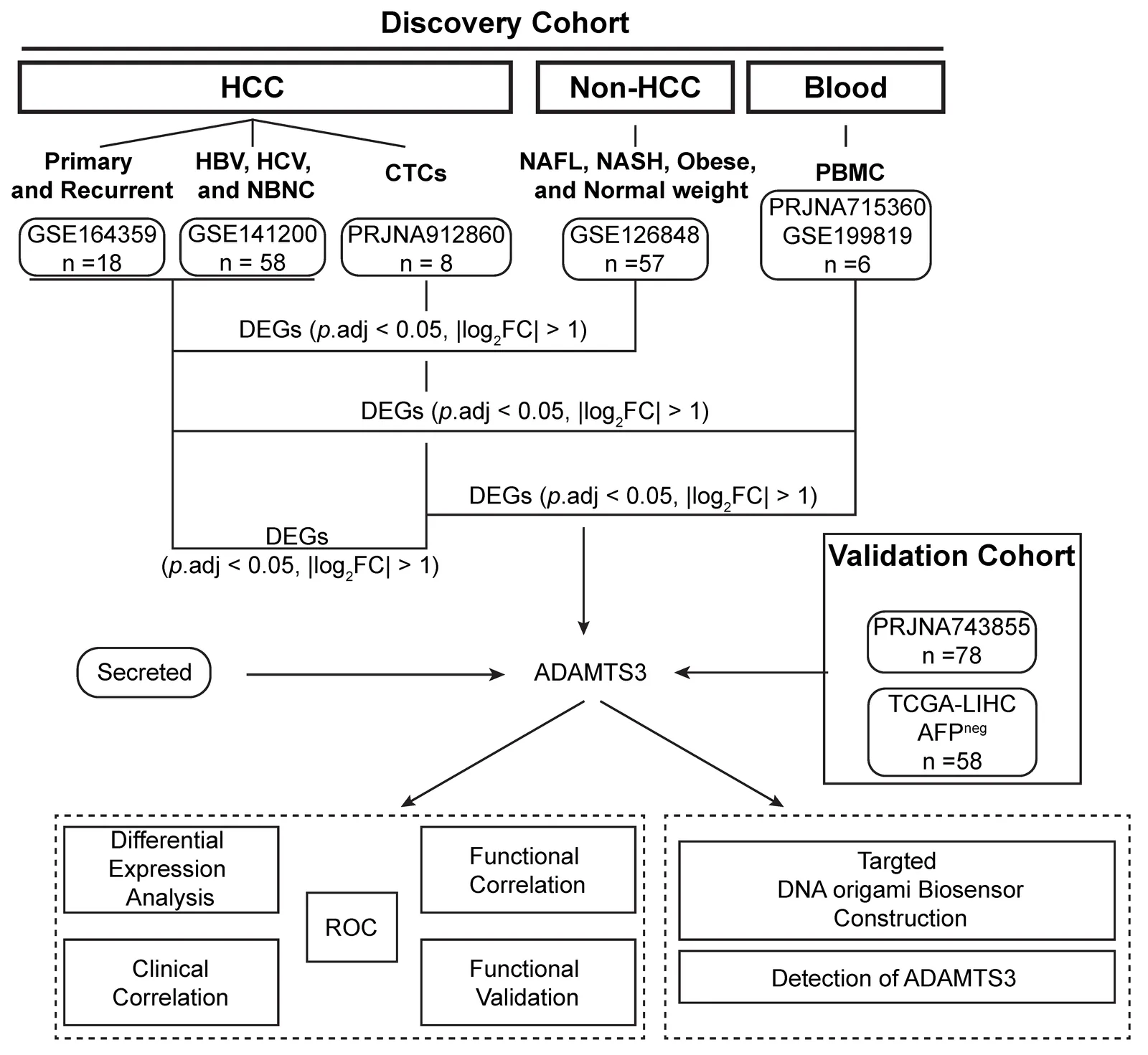
The flowchart of data collection and analysis.

To identify secreted protein candidates associated with HCC detection, we first analyzed molecular profiles from a discovery cohort comprising non-HCC individuals, including healthy controls and patients with obesity or NAFLD, as well as HCC patients with diverse etiologies (Hepatitis B virus, Hepatitis C virus, and non-viral) and disease stages (Figure 1).

**Figure 2:**
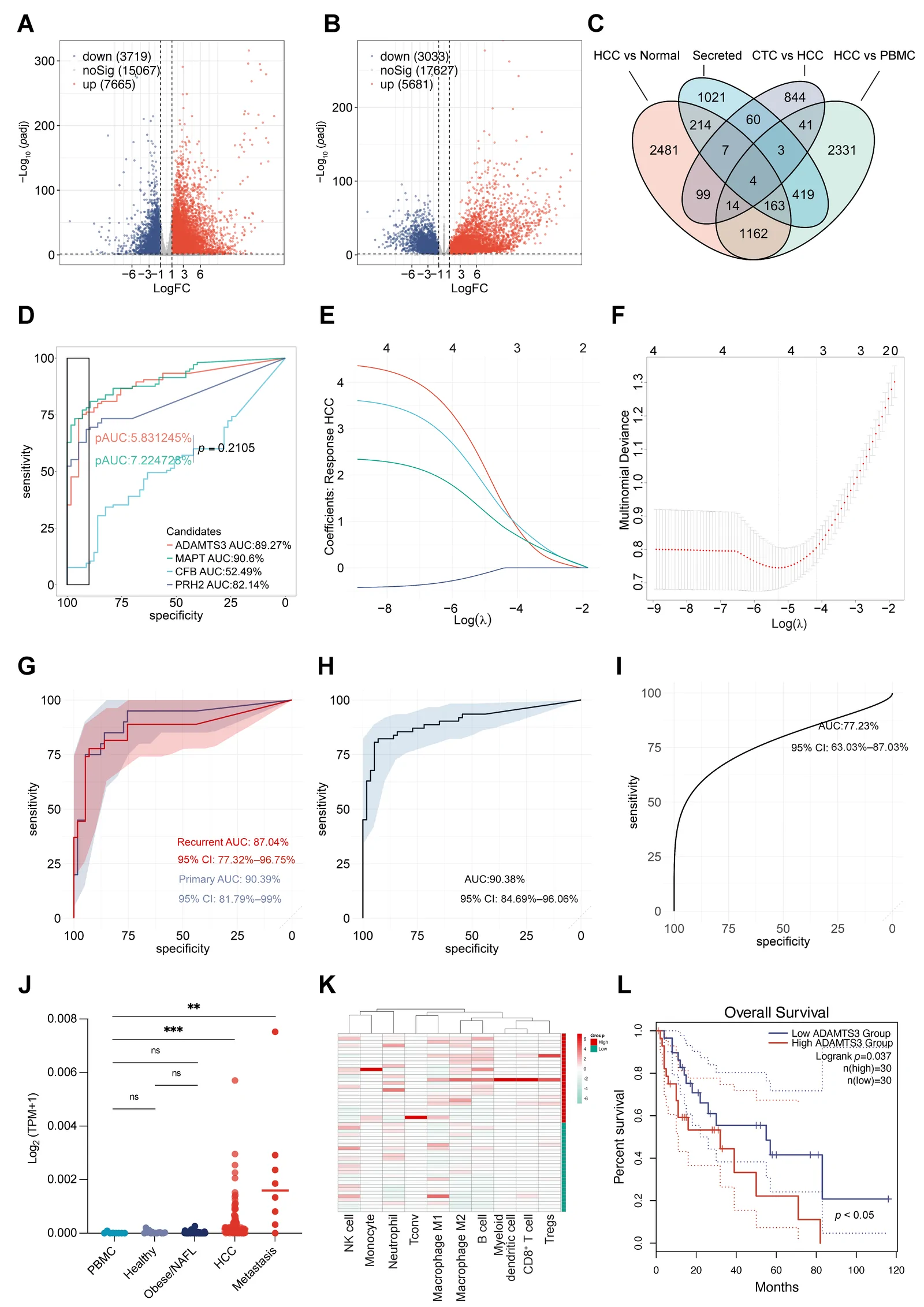
Identification of ADAMTS3 through intersection-based transcriptomic analysis in HCC. (**A**) Volcano plot of differentially expressed genes (DEGs) between bulk liver tissues from HCC and non-HCC patients. The red points indicate upregulated genes while the blue points represent downregulated genes (*p*.adj < 0.05 and |log_2_FC|> 1). The gray points represent genes with no significant difference. (**B**) DEGs between HCC tissues and peripheral blood mononuclear cells (PBMCs). The red points indicate upregulated genes while the blue points represent downregulated genes (*p*.adj < 0.05 and |log_2_FC|> 1). The gray points represent genes with no significant difference. (**C**) Overlap of DEGs across comparisons: HCC vs. Normal, Secreted proteins, CTC vs. HCC, and HCC vs. PBMC. (**D**) ROC (receiver operating characteristic) analysis of overlapped DEGs for distinguishing HCC from healthy individuals. (**E**) Coefficient profiles of shared DEGs derived from LASSO regression. (**F**) Cross-validation curve for the penalty term. (**G**) ROC analysis of ADAMTS3 for distinguishing primary and recurrent HCC from healthy individuals. (**H**) ROC analysis of ADAMTS3 in an independent validation cohort. (**I**) Evaluation of ADAMTS3’s transcriptomic discriminatory ability in HCC patients with AFP-negative HCC cases. (**J**) Relative expression levels of ADAMTS3 in PBMCs, healthy individuals, individuals with obesity or non-alcoholic fatty liver (NAFL) disease, HCC patients, and circulating tumor cells (CTCs) from HCC patients. (**K**) Immune infiltration analysis comparing HCC samples with high (upper quartile) and low (lower quartile) ADAMTS3 expression. (**L**) Kaplan–Meier survival analysis of HCC patients with high versus low ADAMTS3 expression. ns indicates *p*  >  0.05; ** indicates *p*  <  0.01; *** indicates *p*  <  0.001.

We performed differential expression analysis across three independent comparisons to identify genes consistently associated with HCC. A comparison of HCC and non-HCC liver tissues identified 7665 genes as significantly upregulated and 3719 genes as significantly downregulated (Figure 2A). A comparison of HCC tissues with peripheral blood mononuclear cells (PBMCs) identified 5681 upregulated and 3033 downregulated genes (Figure 2B). Additionally, differential analysis comparing circulating tumor cells (CTCs) to HCC tissues identified 1861 upregulated and 287 downregulated genes (Figure S1A). All upregulated genes were further filtered using a threshold of |Log_2_FoldChange| > 1.5, intersected across comparisons, and then cross-referenced with secreted proteins annotated in the GeneCards database. This yielded four candidate genes that are both secreted and consistently upregulated across the discovery cohort (Figure 2C). We further evaluated their transcriptomic discrimination performance for distinguishing HCC from healthy individuals with ROC (receiver operating characteristic) analysis. Among these candidates, ADAMTS3 and MAPT showed the highest area under the curve (AUC) values in ROC analysis (Figure 2D). We also compared the partial AUC within the 90–100% specificity range for ADAMTS3 and MAPT [28]. No significant difference was observed between the two proteins in this high-specificity range (*p* = 0.2105). However, ADAMTS3 showed a stronger contribution within the LASSO model and was selected for subsequent analyses (Figure 2E,F). We assessed the transcriptomic discrimination performance of ADAMTS3 in datasets comprising primary HCC versus healthy controls, as well as recurrent HCC versus healthy controls. Notably, ADAMTS3 retained discriminatory ability, achieving an AUC of 0.9039 (95% CI: 81.79–99%) for primary HCC and 0.8704 (95% CI: 77.32–96.75%) for recurrent HCC (Figure 2G). We also assessed the performance of ADAMTS3 in obesity/NAFL and HCC populations. ADAMTS3 showed significant discriminatory capacity with an AUC of 0.9062 (95% CI: 83.67–95.60%) in distinguishing HCC from obesity and NAFL cases (Figure S1B). To further evaluate the consistency of these findings, we evaluated ADAMTS3 performance across two independent validation cohorts. In a cohort with unspecified AFP status, ADAMTS3 showed an AUC of 0.9038 (95% CI: 84.69–96.06%) (Figure 2H). In another clinically defined AFP-negative HCC cohort, ADAMTS3 also demonstrated transcriptomic discriminatory ability, with an AUC of 0.7723 (95% CI: 63.013–87.03%) (Figure 2I). Further, we analyzed ADAMTS3 expression across different stages of HCC progression. No significant difference was observed between the obesity/NAFL and healthy groups, whereas ADAMTS3 levels were significantly elevated in HCC compared to both healthy and obesity/NAFL groups (Figure 2J). In line with these findings, the upregulation of ADAMTS3 was reproduced in the validation cohort of AFP-negative HCC patients (Figure S1C). ADAMTS3 expression among PBMCs, normal liver tissues, obese/NAFL tissues, HCC tissues, and metastatic samples (CTCs) progressively increased with disease progression (Figure 2J). In addition, mapping molecular features to biological behaviors, we observed a significant enrichment of M2 macrophages and a corresponding exclusion of natural killer (NK) cells in the tumor microenvironment of the high ADAMTS3 group (Figure 2K). Finally, 10-year overall survival was significantly reduced in patients with high ADAMTS3 expression (Figure 2L).

### 3.2. ADAMTS3 Knockdown Impairs HCC Cell Growth and Migration and Induces Apoptosis

**Figure 3:**
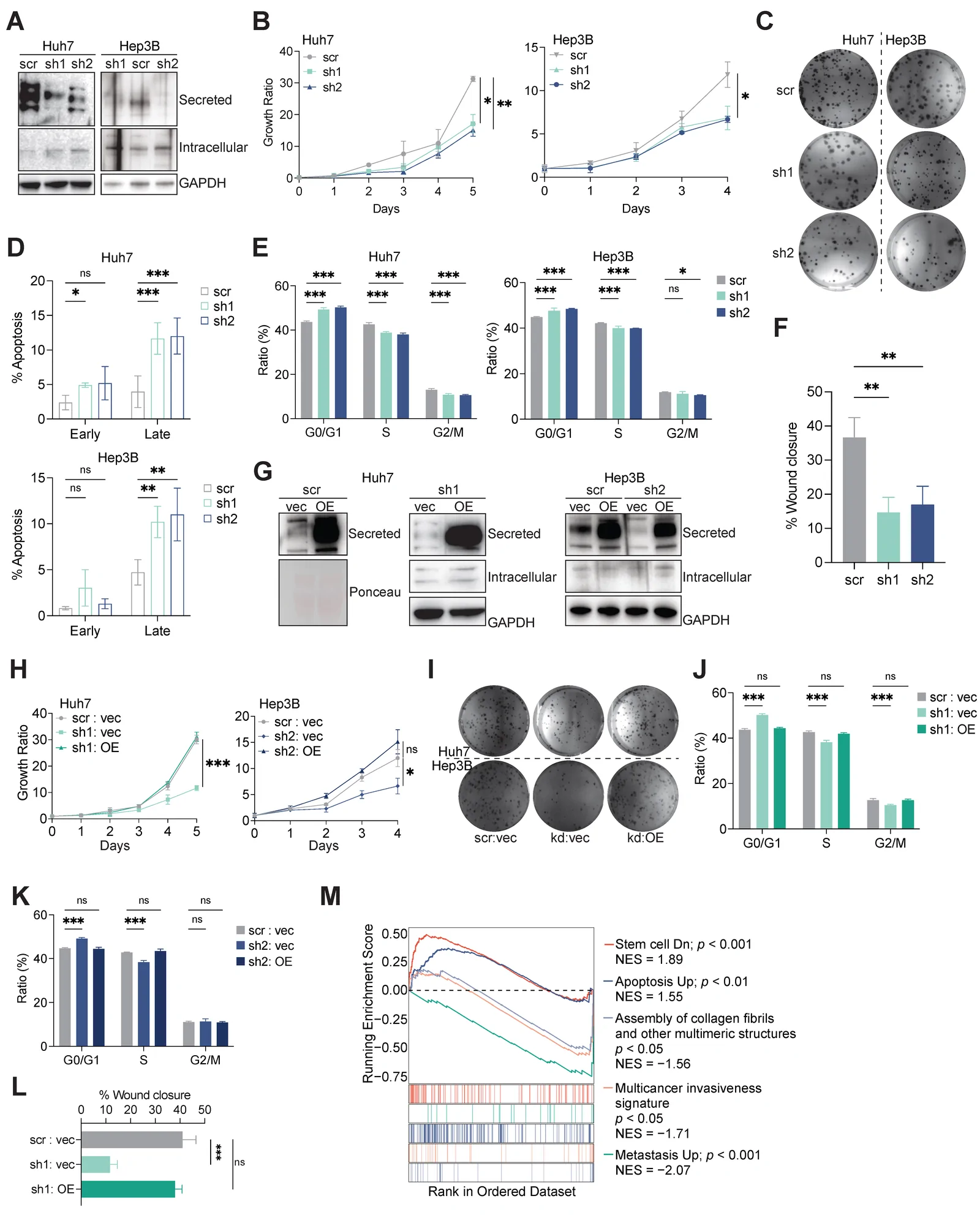
ADAMTS3 knockdown impairs HCC cell growth and migration and induces apoptosis. (**A**) Representative Western blots of ADAMTS3 in wild-type or ADAMTS3 knockdown cells. (**B**) Proliferation curves of wild-type and ADAMTS3 knockdown cells; cells were plated in normal growth medium in 96-well plates and cell viability was measured daily by CCK-8 assay for 4–5 days. (**C**) Colonies of wild-type and ADAMTS3 knockdown cells as indicated. (**D**) Apoptosis assessment by Annexin V/PI dual staining and quantification in both wild-type and ADAMTS3 knockdown cells. (**E**) Cell-cycle distribution in indicated cell lines assessed by flow cytometry. (**F**) Quantification of wound closure rate after 12 h in the indicated Huh7 cells. (**G**) Representative Western blots of ADAMTS3 in ADAMTS3 knockdown and scramble control cells introduced either vector or ADAMTS3. (**H**) Proliferation curves of indicated cells; cells were transduced with vector or ADAMTS3 expression constructs, plated in normal growth medium in 96-well plates, and analyzed by CCK-8 assays. (**I**) Colonies of ADAMTS3 knockdown cells introduced either vector or ADAMTS3. (**J**,**K**) Cell-cycle distribution of Huh7 (**J**) and Hep3B (**K**) cells in the ADAMTS3-knockdown background with or without ADAMTS3 re-expression assessed by flow cytometry. (**L**) Quantification of wound closure rate after 12 h in the indicated Huh7 cells. (**M**) GSEA of pathways altered by ADAMTS3 knockdown. Results presented are representatives from three independently conducted experiments. Growth curves, apoptosis, and cell-cycle distribution were analyzed using two-way ANOVA followed by Tukey’s multiple comparisons test. Other statistical comparisons were performed using two-tailed Student’s *t*-tests. ns indicates *p*  >  0.05; * indicates *p*  <  0.05; ** indicates *p*  <  0.01; *** indicates *p*  <  0.001.

To better delineate the functional effects of ADAMTS3 in HCC cells, we generated ADAMTS3-knockdown Huh7 cell lines to assess its impact on cellular behaviors and related molecular changes (Figure 3A, Table 1). Knockdown of ADAMTS3 led to significantly reduced cell proliferation (Figure 3B and Figure S2A). Further colony formation assays demonstrated impaired clonogenic capacity in ADAMTS3 knockdown cells, indicating that ADAMTS3 contributes to both proliferative and clonogenic potential in HCC cells (Figure 3C). Of note, ADAMTS3 knockdown increased apoptotic cell death (Figure 3D and Figure S2B,C) and induced G1 cell-cycle arrest (Figure 3E and Figure S2D), which likely contributed to the impaired growth of ADAMTS3 knockdown cells. Moreover, ADAMTS3 knockdown significantly impaired cell migration, as demonstrated by reduced wound closure rates (Figure 3F and Figure S2E).

We next performed rescue experiments by restoring ADAMTS3 expression in knockdown cells to further confirm the functional contribution of ADAMTS3 (Figure 3G). Reintroduction of ADAMTS3 effectively reversed the proliferation inhibition (Figure 3H) and partially rescued clonogenic ability (Figure 3I). In addition, ADAMTS3 re-expression alleviated G1 cell-cycle arrest (Figure 3J,K and Figure S2F) and enhanced migratory capacity (Figure 3L and Figure S2G). These findings further support a functional role for ADAMTS3 in regulating HCC cell proliferation, clonogenicity, cell-cycle progression, and migration in vitro.

We further performed RNA-seq analysis to characterize the transcriptional changes induced by ADAMTS3 knockdown (Figure S2H). Gene ontology analysis revealed enrichment of extracellular matrix components, including the collagen-containing extracellular matrix and basement membrane, as well as the external side of the plasma membrane (Figure S2I). Further analysis of tumor-related gene signatures revealed that ADAMTS3 knockdown was associated with enhanced apoptotic, together with reduced signatures of invasiveness and metastasis, indicating a marked decrease in tumorigenic potential (Figure 3M). In summary, ADAMTS3 knockdown suppresses the malignant phenotypes of HCC cells in vitro, characterized by impaired cell growth and clonogenicity, cell-cycle arrest, and reduced migratory capacity.

### 3.3. TGF-β:TGFBR2 Signaling Is Involved in ADAMTS3 Knockdown-Induced Effects on HCC Cell Growth and Migration

**Figure 4:**
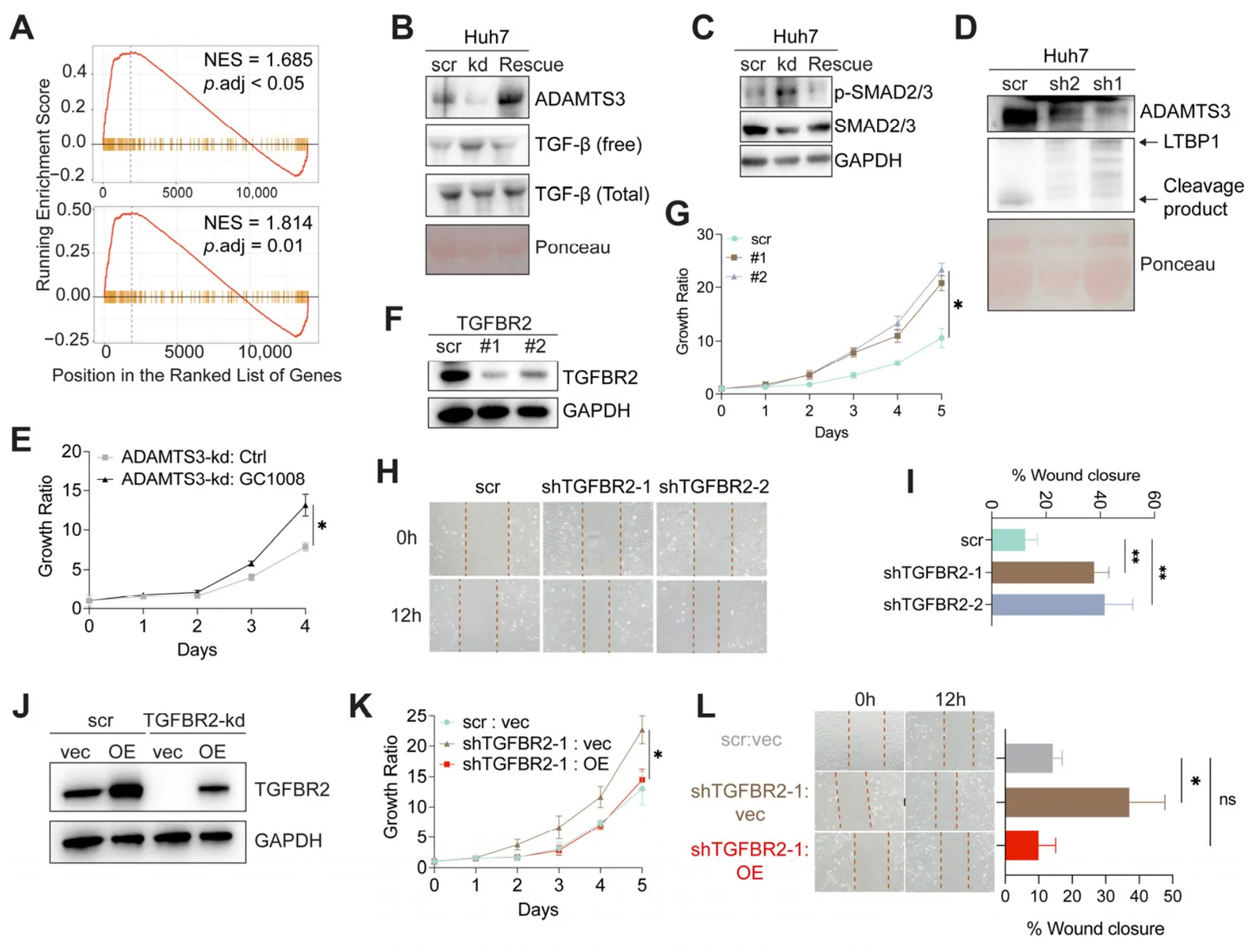
TGF-β:TGFBR2 signaling is involved in ADAMTS3 knockdown-induced effects on HCC cell growth and migration. (**A**) GSEA of TGF-β signaling after ADAMTS3 knockdown. (**B**) Representative Western blots of ADAMTS3 and TGF-β in indicated cells. (**C**) Levels of p-SMAD2/3 (Ser465/467 of SMAD2; Ser423/425 of SMAD3) and total SMAD2/3 assessed by Western blotting in the indicated cells. (**D**) Representative Western blots of ADAMTS3 and LTBP1 in scramble or ADAMTS3-knockdown Huh7 cells. (**E**) Proliferation curves of ADAMTS3-knockdown Huh7 cells treated with or without TGF-β-neutralizing antibody (GC1008). (**F**) Representative Western blot of TGFBR2 in scrambled control and TGFBR2-knockdown cells in the ADAMTS3-knockdown Huh7 background. (**G**–**I**) Proliferation curves(**G**) and wound closure rate (**H**,**I**) of ADAMTS3-knockdown Huh7 cells with or without TGFBR2 knockdown. (**J**) Representative Western blot of TGFBR2 expression in ADAMTS3-knockdown Huh7 cells with TGFBR2 knockdown or re-expression. (**K**,**L**) Proliferation curves (**K**) and wound closure rate (**L**) of ADAMTS3-knockdown Huh7 cells following TGFBR2 knockdown or re-expression. ns indicates *p*  >  0.05; * indicates *p*  <  0.05; ** indicates *p*  <  0.01.

Although N-proteinase ADAMTS3 has been primarily characterized for its role in collagen processing, accumulating evidence indicates that it also cleaves non-collagenous substrates, including LTBP1 and VEGF-C, thereby regulating diverse physiological processes [35,36,37]. Based on transcriptomic profiling of ADAMTS3-knockdown Huh7 cells and their corresponding scramble controls, we further investigated the downstream signaling pathways regulated by these substrates, with a particular focus on VEGF and LTBP1-associated TGF-β signaling. Interestingly, ADAMTS3 knockdown preferentially induced TGF-β stimulation signature (Figure 4A). In line with this, ADAMTS3 knockdown increased extracellular TGF-β levels, accompanied by the enhanced phosphorylation of its canonical downstream SMAD2/3 (Figure 4B,C).

However, ADAMTS3 knockdown also impaired LTBP1 processing (Figure 4D). LTBP1 associates with latent TGF-β complexes and helps localize them to the extracellular matrix, where it can facilitate regulated TGF-β activation, including through integrin-mediated mechanisms [38]. Thus, the reduced LTBP1 processing observed following ADAMTS3 knockdown suggesting that the relationship between ADAMTS3-dependent LTBP1 processing and TGF-β signaling may be context-dependent.

To determine whether the increased extracellular TGF-β contributed to the cellular effects of ADAMTS3 knockdown, we first neutralized extracellular TGF-β using a pan-TGF-β-neutralizing antibody. Neutralization of extracellular TGF-β alleviated the growth inhibition induced by ADAMTS3 knockdown (Figure 4E). Given that TGFBR2 is essential for canonical TGF-β signal transduction [39], we further generated TGFBR2-knockdown cells in the ADAMTS3-knockdown background to determine whether TGFBR2 contributes to the tumor-suppressive response triggered by ADAMTS3 depletion (Figure 4F). The reduction in cell growth induced by ADAMTS3 knockdown was partially rescued by TGFBR2 knockdown (Figure 4G). Similarly, TGFBR2 knockdown accelerated wound closure, indicating restored migratory capacity (Figure 4H,I). Consistently, further re-expression of shRNA-resistant TGFBR2 restored the inhibitory effects of ADAMTS3 knockdown on both cell growth and migration (Figure 4J–L).

Taken together, these findings support the involvement of TGF-β:TGFBR2 signaling in the cellular effects of ADAMTS3 knockdown.

### 3.4. Screening and Validation of an ADAMTS3-Targeting Aptamer for DNA Origami-Based SERS Biosensing

**Figure 5:**
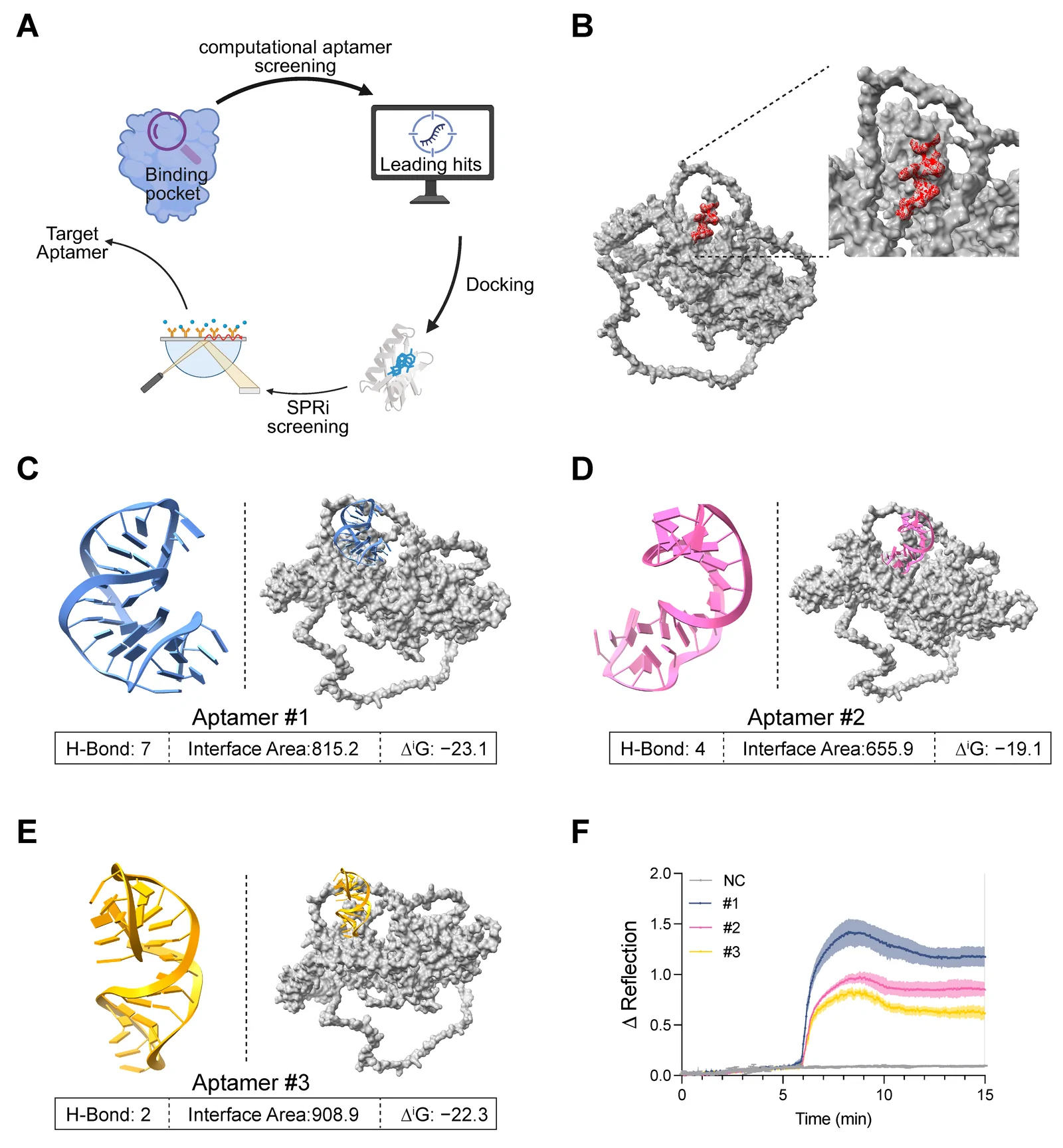
Screening and validation of an ADAMTS3-targeting aptamer for DNA origami-based SERS biosensing. (**A**) Workflow of in silico screening and experiment validation of candidate aptamers. (**B**) Binding pocket of ADAMTS3 for aptamer targeting. (**C**) Visual representation of Aptamer #1-ADAMTS3 docked complex. (**D**) Visual representation of Aptamer #2-ADAMTS3 docked complex. (**E**) Visual representation of Aptamer #3-ADAMTS3 docked complex. (**F**) Experimental validation of the computational predicted candidate aptamers with SPRi.

Motivated by these findings, we aimed to develop a ADAMTS3-targeting aptamer. To this end, we employed an in silico screening pipeline to identify high-affinity candidate aptamers targeting the ADAMTS3 binding pocket, which were subsequently evaluated by surface plasmon resonance imaging (SPRi) assay (Figure 5A). Using the pocket detection algorithm CASTpFold [24], we identified the N-terminal region of ADAMTS3 as the most favorable site for aptamer binding (Figure 5B). Based on this binding pocket, an in silico screening of aptamers was performed with AptaLoop pipeline [40]. The Top3 leading hits were selected for further evaluation of their intermolecular interactions with ADAMTS3 using HADDOCK docking and PDBePISA, in which residues within the predicted ADAMTS3 binding pocket were defined as active residues for docking [26].

All three aptamers exhibited a favorable binding tendency toward ADAMTS3, yet their predicted interaction profiles varied. Aptamer #1 appeared to engage the protein most extensively, forming 7 hydrogen-bonds (H-bond) and covering an interface area of 815.2 Å^2^, with a predicted interface free energy (∆^i^G) of −23.1 kcal/mol (Figure 5C). In comparison, Aptamer #2 formed fewer contacts, establishing only 4 H-bonds with an interface area of 655.9 Å^2^ (∆^i^G: −19.1 kcal/mol) (Figure 5D). Interestingly, Aptamer #3, while forming only 2 H-bonds, exhibited a larger interface area of 908.9 Å^2^ (∆^i^G: −22.3 kcal/mol), suggesting a distinct binding mode (Figure 5E). Collectively, these results indicate that the three aptamers exhibit different interaction patterns with ADAMTS3, providing a rationale for subsequent SPRi experiments to directly compare their binding responses.

Further SPRi assays revealed that Aptamer #1 generated the highest ΔReflection signal, indicating the strongest binding response, whereas Aptamers #2 and #3 showed moderate and lower signals, respectively (Figure 5F). More important, Aptamer #1 showed preferential binding to ADAMTS3, with weaker binding to the structurally similar family member ADAMTS2 and to Albumin (Figure S3A).

SERS offers a highly sensitive biosensing approach by leveraging the ability of metallic nanostructures to tightly confine electromagnetic fields, thereby enhancing Raman vibrational signals [41]. We designed a SERS-based immunosensor for detection of ADAMTS3. As shown in Figure S3B, this sensor utilizes a DNA origami scaffold to precisely position two gold nanospheres conjugated with Aptamer#1 into a dimer, creating a single SERS hotspot that enables high sensitivity, potentially down to the single-molecule level [18]. Unlike probes based on randomly distributed nanoparticles [42], this site-specific arrangement ensures the aptamer is located exactly within the SERS hotspot for optimal target binding.

Transmission electron microscopy (TEM) confirmed the expected dimer configuration with a narrow interparticle gap (Figure S3C). Functionalized with Aptamer #1, the sensor detected purified ADAMTS3 protein across a range of concentrations, demonstrating a sensitivity down to 10^−12^ M (Figure S3D). The Raman peak at 1620 cm^−1^, a signature signal of the protein’s protein β-sheet structure, intensified progressively with increasing ADAMTS3 concentration. Accordingly, we focused our quantitative analysis on this characteristic peak. We focused on this signal to analyze the concentration intensity response. The relative Raman intensity of the protein signal at 1620 cm^−1^, normalized to the internal DNA reference signal at 1095 cm^−1^, showed strong linear response with R^2^ = 0.914, highlighting the sensor’s quantitative capability (Figure S3E).

In summary, this DNA origami-enabled SERS aptasensor offers a sensitive, and quantitative platform for the selective detection of ADAMTS3 protein, supporting further exploration of ADAMTS3-based assessment approaches.

## 4. Conclusions

The shifting landscape of HCC etiology highlights the need for complementary approaches to improve HCC detection [6]. In this study, we sought to identify secreted proteins for further investigation by examining transcriptomic profiles associated with emerging HCC risk factors, including alcohol-associated liver injury and obesity. Through intersection-based analysis of transcriptomic profiles from HCC tissues and non-HCC liver samples representing diverse clinical backgrounds, we prioritized ADAMTS3 for further study. Although the diagnostic performance of serum ADAMTS3 protein remains unknown, our findings indicate that ADAMTS3 is involved in the regulation of HCC cell growth and migration through the TGF-β:TGFBR2 axis in vitro. Additionally, we developed a corresponding aptamer for DNA origami-based SERS-biosensing, which provides a potential platform for future protein-level evaluation on ADAMTS3.

Based on transcriptomic data, ADAMTS3 showed an AUC of 0.9039 (95% CI: 81.79–99.00%) for distinguishing primary HCC and 0.8704 (95% CI: 77.32–96.75%) for recurrent HCC. Across two validation cohorts, ADAMTS3 transcript levels showed an AUC of 0.7723 (95% CI: 63.03–87.03%) in AFP-negative HCC cases and 0.9037 (95% CI: 84.69–96.06%) in cases with unspecified AFP status. Importantly, these findings are derived solely from transcriptomic analyses, and the performance of ADAMTS3 at the protein level in serum remains to be established.

Despite the transcriptomic evidence linking ADAMTS3 to HCC, its biological function and molecular mechanism in HCC remain insufficiently characterized [14]. Therefore, we first investigated the functional effects of ADAMTS3 on HCC cells in vitro. Functional studies demonstrated that ADAMTS3 knockdown markedly impaired HCC cell proliferation and migration, accompanied by enhanced apoptosis and G1 cell-cycle arrest, whereas ADAMTS3 re-expression partially rescued these effects. These findings support a functional role for ADAMTS3 in regulating HCC cell proliferation, migration, apoptosis, and cell-cycle progression in vitro. Further transcriptomic profiling revealed preferential activation of TGF-β-related transcriptional programs following ADAMTS3 knockdown. In line with this, extracellular TGF-β levels were increased, accompanied by enhanced phosphorylation of downstream SMAD2/3. Neutralization of extracellular TGF-β alleviated the growth inhibition induced by ADAMTS3 knockdown. As a key receptor for TGF-β signaling, TGFBR2 knockdown partially rescued the impaired cell growth and migration induced by ADAMTS3 knockdown, whereas TGFBR2 re-expression restored these inhibitory effects.

TGF-β signaling regulates cell growth, migration, and death, with its effects in cancer being highly context-dependent [43]. In HCC, previous studies have shown that TGF-β can suppress tumor initiation but acquire tumor-promoting effects during tumor progression [39,44,45]. Elevated TGF-β expression has also been associated with adverse pathological features and poor prognosis in HCC, while its activity has been linked to immune-cell exhaustion and M2 macrophage differentiation within the tumor microenvironment [44,46]. In contrast to these studies, which have largely examined the role of TGF-β in HCC directly, our findings identify ADAMTS3 as an upstream factor associated with TGF-β signaling in HCC cells.

Intriguingly, given the reported role of ADAMTS3 in LTBP1 cleavage and the involvement of LTBP1 in integrin-dependent TGF-β activation [14,35,37,47,48], we observed reduced processing of pro-LTBP1 upon ADAMTS3 knockdown, suggesting the relationship between LTBP1 processing and TGF-β signaling may also be context-dependent. Further studies will be needed to define how ADAMTS3-dependent extracellular matrix remodeling is integrated with TGF-β signaling in HCC cells.

Taken together, our findings prioritize ADAMTS3 for further investigation in HCC and support its involvement in the regulation of HCC cell growth and migration in vitro. The observed changes in TGF-β signaling and the rescue effects of TGFBR2 manipulation suggest that TGF-β:TGFBR2 signaling is involved in the cellular effects associated with ADAMTS3 knockdown. In parallel, we developed a DNA origami-based SERS biosensing approach for ADAMTS3 detection, providing an analytical platform for future protein-level studies. Further evaluation will be required to determine whether circulating or serum ADAMTS3 protein has diagnostic value in HCC.

## Figures and Tables

**Table 1:** Key resources table.

Reagent or Resource	Source	Identifier
Antibodies		
Mouse monoclonal anti-ADAMTS3	Santa Cruz Biotechnology (Dallas, TX, USA)	Cat# sc-517029;
Mouse monoclonal anti-GAPDH	Proteintech (Rosemont, IL, USA)	Cat# 60004-1-Ig; RRID:AB_2107436
HRP-conjugated goat polyclonal anti-mouse IgG	Huabio (Hangzhou, China)	Cat# HA1006; RRID:AB_2819167
Rabbit polyclonal anti-LTBP1	Proteintech	Cat# 26855-1-AP; RRID:AB_2880658
Rabbit polyclonal anti-SMAD2/3	Proteintech	Cat# 12570-1-AP; RRID:AB_2193037
Rabbit monoclonal anti-Phospho-SMAD2 (Ser465/467)/SMAD3 (Ser423/425)	Proteintech	Cat# 80427-2-RR
Rabbit monoclonal anti-TGF Beta 1	Proteintech	Cat# 81746-2-RR
Mouse monoclonal anti-TGFBR2	Proteintech	Cat# 66636-1-Ig; RRID:AB_2881995
Chemicals, peptides, and recombinant proteins		
EDTA-free Protease Inhibitor Cocktail Tablets	Sigma-Aldrich	Cat# 05056489001
GC1008 (monoclonal antibody against TGF-β)	MCE (Monmouth Junction, NJ, USA)	Cat# HY-P99020
Critical commercial assays		
BeaverBeads™ His-tag Protein Purification Kit	Beaver (Suzhou, China)	Cat# 70501
Deposited data		
RNA-seq data: PBMC	ENA	PRJNA715360; PRJNA821540
RNA-seq data: Liver biopsies of HCC	GEO & ENA	GSE164359; GSE141200; PRJNA912860; PRJNA743855
RNA-seq data: Huh7 ADAMTS3kd vs. Scramble	This study	
RNA-seq data: Liver biopsies of NAFL, NASH, Obese, and Healthy subjects	GEO	GSE126848
Experimental models: Cell lines		
Huh7	Immocell	IM-H040
HEK-293T	ATCC	CRL-3216
Huh7 ADAMTS3 Kd cells	This study	N/A
Huh7 3FlagADAMTS3 cells	This study	N/A
Huh7 scramble cells	This study	N/A
Huh7 ADAMTS3&TGFBR2 Kd cells	This study	N/A
Huh7 ADAMTS3 Kd & TGFBR2 cells	This study	N/A
Oligonucleotides		
Oligonucleotides	This study	Table S1
Recombinant DNA		
piggyBac-EF1a-3xFLAG-ADAMTS3-IRES-Puro	This study	N/A
piggyBac-EF1a-3xHis-ADAMTS3-IRES-Puro	This study	N/A
ADAMTS3 knockdown shRNA	This study	N/A
TGFBR2 knockdown shRNA	This study	N/A
piggyBac-EF1a-TGFBR2-IRES-Puro	This study	N/A
Software and algorithms		
R v4.4.1	R Foundation	https://cran.r-project.org/bin/macosx/, accessed on 16 September 2024.
DESeq2 v1.44.0	Love et al. [29]	https://bioconductor.org/packages/release/bioc/html/DESeq2.html, accessed on 16 September 2024.
ggplot2 v3.5.1	H. Wickham [30]	https://cran.r-project.org/web/packages/ggplot2/index.html, accessed on 16 September 2024.
clusterProfiler v4.12.2	Yu et al. [31]	https://bioconductor.org/packages/release/bioc/html/clusterProfiler.html, accessed on 16 September 2024.
pROC v1.18.5	Robin et al. [28]	https://xrobin.github.io/pROC/, accessed on 16 September 2024.
Msigdbr v7.5.1	Igor Dolgalev [32]	https://cran.r-project.org/web/packages/msigdbr/index.html/, accessed on 16 September 2024.
Tximeta v1.22.1	Love et al. [33]	https://github.com/thelovelab/tximeta/, accessed on 22 October 2024.
Graphpad Prism 10	Graphpad	https://www.graphpad.com/, accessed on 22 July 2024.
glmnet v4.1-10	Friedman et al. [34]	https://cran.r-project.org/web/packages/glmnet/index.html, accessed on 19 July 2025.

## Data Availability

The raw data supporting the conclusions of this article will be made available by the authors on request.

## Supplementary Materials

The following supporting information can be downloaded at: https://www.mdpi.com/article/10.3390/cells15191784/s1, Figure S1 related to Figure 2: A differential analysis comparing circulating tumor cells to HCC tissues. B ROC analysis of ADAMTS3 in distinguishing HCC from obesity and NAFL cases. C ADAMTS3 expression level in validation cohort; Figure S2 related to Figure 3: A Growth curve of Huh7 and Hep3B based on cell counting. Cells were counted every 24 h after seeding. B Representative Annexin V/PI staining profiles showing apoptotic cell populations following ADAMTS3 knockdown. C Quantification of overall apoptotic rate following ADAMTS3 knockdown. D Representative flow cytometry profiles showing cell cycle distribution of Huh7 and Hep3B cells following ADAMTS3 knockdown. E Representative images of wound closure assays using Huh7 cells with or without ADAMTS3 knockdown. F Representative flow cytometry profiles showing cell cycle distribution of Huh7 and Hep3B cells in the ADAMTS3-knockdown background with or without ADAMTS3 re-expression. G Representative images of wound closure assays using ADAMTS3-knockdown Huh7 cells re-expression of ADAMTS3 or vector. H Volcano plot showing differentially expressed genes upon ADAMTS3 knockdown. I GO analysis on differentially expressed genes upon ADAMTS3 knockdown. Figure S3 related to Figure 5: A Binding responses of Aptamer #1 to ADAMTS3, ADAMTS2, and albumin. B Numerical simulation illustrating the electromagnetic field confinement/enhancement between the AuNPs. C TEM micrograph of two Au NPs self-assembled on the origami structure. D Normalized intensity of ADAMTS3 at varying concentrations, measured on the DNA origami scaffold functionalized with Aptamer #1. E Scatter plot of the normalized intensity, defined as the ratio of the protein signal at 1620 cm^−1^ to the internal DNA reference signal at 1095 cm^−1^, versus the concentration of ADAMTS3.Table S1: Sequences of oligonucleotides used in this study.
