# Supplementary material for: ADAMTS3 Promotes Hepatocellular Carcinoma Cell Growth and Migration Through TGF-β:TGFBR2 Signaling
Source: Cells. 2026 Sep 30;15(19):1784. doi: 10.3390/cells15191784 (PMC13664179; doi:10.3390/cells15191784)
Supplement: Supplementary file 1 [file cells-15-01784-s001.zip › Figure S1.pdf]

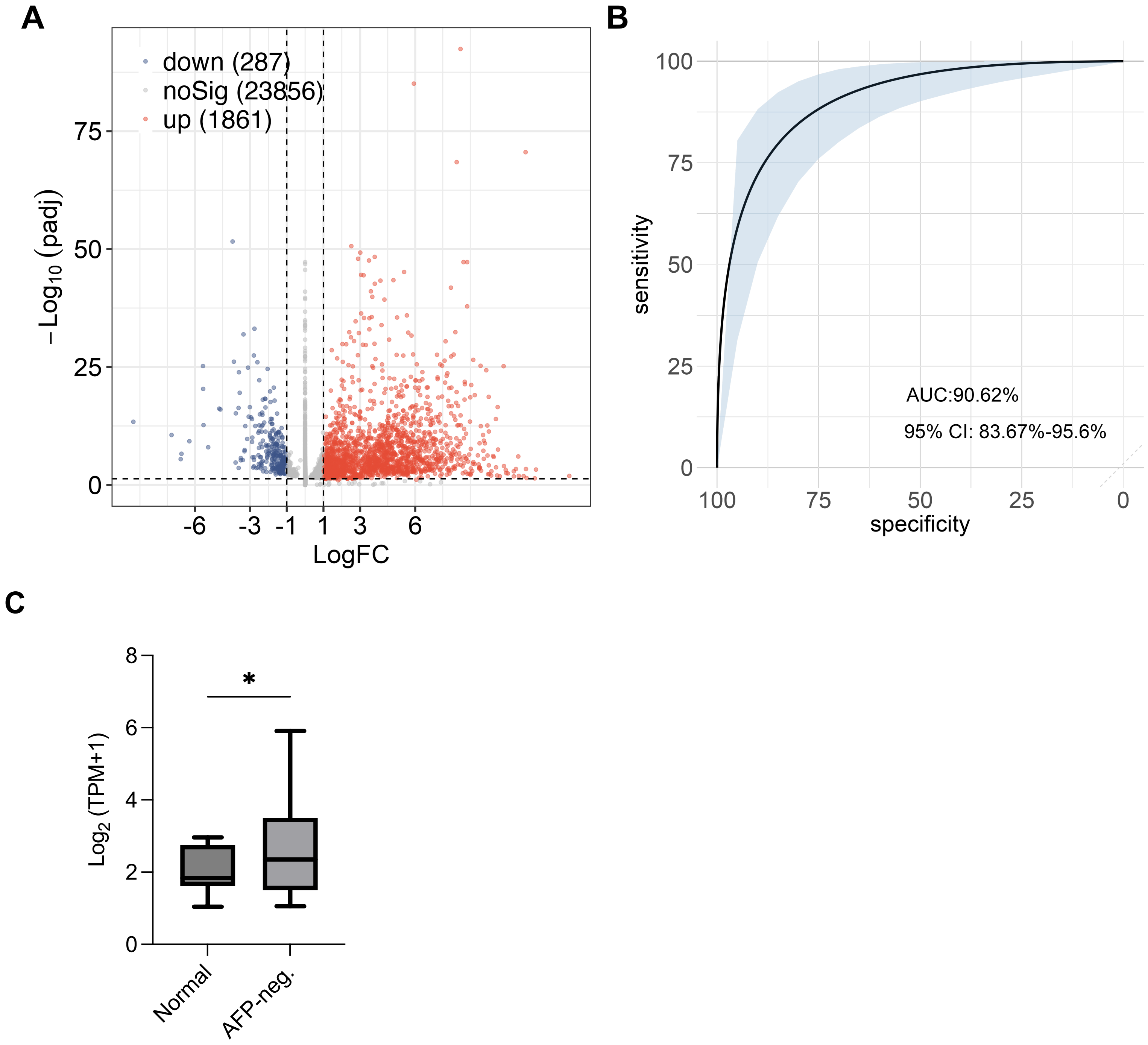

A
down (287)
noSig (23856)
up (1861)
−Log10 (padj)
LogFC
B
sensitivity
specificity
AUC:90.62%
95% CI: 83.67%-95.6%
C
Log2 (TPM+1)
*
Normal
AFP-neg.
