# Supplementary material for: ADAMTS3 Promotes Hepatocellular Carcinoma Cell Growth and Migration Through TGF-β:TGFBR2 Signaling
Source: Cells. 2026 Sep 30;15(19):1784. doi: 10.3390/cells15191784 (PMC13664179; doi:10.3390/cells15191784)
Supplement: Supplementary file 1 [file cells-15-01784-s001.zip › Table S1.pdf]

**Aptamers**	**Sequence (5'-3')**
Aptamer #1	GUAGCGGGAGAGGCCGCUCCGAAUGAAA
Aptamer #2	GUAGGGGGAGGGACCCCUACGAAUGAAA
Aptamer #3	GUAGCGGGAGUCGCCCCAACGAAUGAAA
**shRNA**	**Target Sequence (5'-3')**
shTGFBR2-1	GACCTCAAGAGCTCCAATATC
shTGFBR2-2	CGACATGATAGTCACTGACAA
shADAMTS3-1	GCAGACTAACTGTGCTTATGT
shADAMTS3-2	GCATGTGGAAGAATGCTAATC
**Primers for expression vectors**	**Sequence (5'-3')**
Fwd-flag	accggcgcctactctagagctagcgaattcgccaccatggactacaaagacc
Rev-flag	aaccaaagtgacaggagaaccatggatcccttgtcatcgtcatccttgtaa
Fwd-ADA	atggttctcctgtcactttggttg
Rev-ADA	ggggagggagaggggcgcggccgcttatctttctaaggtggatgatc
SilentMut1-ADA-R	acatatgcacaattcgtttgcaaagg
SilentMut1-ADA-F	gcaaacgaattgtgcatatgttggtgaca
SilentMut2-ADA-F	gtatgtggaaaaacgcaaaccagc
SilentMut2-ADA-R	ggtttgcgtttttccacatacaatgac
SilentMut-TGFBR2-F	gatctaaaatccagcaacatcctc
SilentMut-TGFBR2-R	gatgttgctggattttagatccctg
Rev-Flag-TGFBR2	gacccatggatcccttgtcatcgtcatc
Fwd-TGFBR2	gacaagggatccatgggtcgggggctgctc
Rev-TGFBR2	gggagggagaggggcgcggccgcggatccttatttggtagtgtttagggagc

Origami-related Sequence (5'-3):

Core staple1 AAGGGACTGGCGCGTCGCCGCGAGCGAGAAAGGA

Core staple2 TGACCTCCGCTACAGTAGCCGGTCAAGA

Core staple3 TTCTGCGAAGAAAAATAACAGAGGTGAGGCCCATTTACATTG

Core staple4 CTACAATAATGAGTGACCCTGATTAGAGCCCGTAAT

Core staple5 TTCATTGCAATACTTTTAGCGAAAATCACC

Core staple6 AACCGGCCTCAGGAAGATCCGTTGTAA

Core staple7 ATAGAAACCAGGCAAAGCGAAAGGGGG

Core staple8 GTCATAGAATGGGATAGGTCACGTTGGTCGTAATCA

Core staple9 AGCCCCCGATAACAGTATGAGCAATTTTATTTTC

Core staple10 GTTAGCGTAACGACGTTGAAA

Core staple11 AACAAGCAAGCCAGAGCCT

Core staple12 GGAACGGTACGTCTGTCGATTGTTCCAGAAG

Core staple13 ATAAGAGCAAGAAAAATAATAATGTACCGACAGTC

Core staple14 TTATCAACAATATTGTTTA

Core staple15 TTAGTACCGACTTTATACATTTCACGACCAGTAATATACCGAACCTCAAA

Core staple16 AACGACGAGCGCTGCGCAGCGAAATTGACGGTAAA

Core staple17 ATTAATGAATCCGGAATAAATACCCATCCTAATT

Core staple18 GAACAACGTATCGGAGGGAGTGAGGCT

Core staple19 GCTGCAAGCGACGTGCTTTC

Core staple20

GCAAAAGAAATACCCAAACAAGAGGATTTAGAAGTTGCTGAACGAACCA
Core staple21 AGCTGGCGCCAAGTGTTTTT
Core staple22 GCAAATCAGATATCTTACC
Core staple23 AGAATAACATAAAATTCTGTCACAACATCACTGCC
Core staple24 ATAATAACTCCAAAGCGGGATACGAGGGTAGCA
Core staple25 GAGCGGACGAACGTACCGCCAAACCCTTCTGACCTTTAATGCAATCAAC
Core staple26 CGCATAGGGTCAATGTATTAAACCAGGC
Core staple27 CGAGTGTACACGGATATATTCAGTAATCATTTCATTATAACAACATTTA
Core staple28 AACCTCCCGACTTTGCACC
Core staple29 AGCGAACGGCCGGACCGGTTGAAATTCG
Core staple30 AACATCACAATATTTATTAATCTAACAACTAATAGGTTGGCAGCGAACT
Core staple31 CCCGCCACCACATAATCTCAGACTGACAGAAGAGCCATGGCTTAATTCG
Core staple32 CATTAAATTTTTGTCAATCATACGGAATGGTGGCA
Core staple33 TCCATCAAACGGAGAGCTGGAGCTAATTCGCGTCTGG
Core staple34 GGTAACGCCGGCGCTAGGGC
Core staple35 AAAATAATCATAGCTTCCTTAGAAAAACCGCTAA
Core staple36 TATTAAACCAAGCAGTTAC
Core staple37 TAACCTTATTAACAAAGCTGCTCTCATTACTTTAACGTATTTCGGA
Core staple38 CTTAAACCATTTCAACAGTTTCAGCGGATTCGAGGTATTCGGTTTTTCA
Core staple39 CGCCACCCTCAGACTAAAGTAACAACCTACCGAT
Core staple40 AAGAAGTGCAACACCGCCTCCCTCAGATTATTAGCTCGG
Core staple41 GAAAGGGGACGACGACAGTTGCCAAGC
Core staple42 TATCAAATACATTTTTCGACAATACTCAGGAGGT
Core staple43 TAGGAACGCTGAATGGCTTGCTTTATCAGCTTGCTGTGAGAATTCTGTA
Core staple44 CCAGCAGCCAGTCATGACGCTCTAAAACTACCAAGTCGCCTGGAAC
Core staple45
CCAGTATATCACCAAAAATCAAGAAAACGAGAATGTCCACGCCATTCAAA
Core staple46 AGAAGAGCGTCGCTGGCCCACGGGTCGA
Core staple47 GCTCATTTTTTAACGTAATCGGCATGATCGTAGAA
Core staple48 ATAAGGGCCGGAAACTTAGTTAGTTGCAGGAAAATC
Core staple49 CCTCAGAACCCACCAGACTAACGGACCAGTC
Core staple50 GAAAAACAAATCAAGGTAAATTTATACATTTAACAAAAAGGTTGAGG
Core staple51 GCATCTATCACACTATTAGCCCGAATG
Core staple52 ACGTCAAAAATGAACGCGCCT
Core staple53 GAAAAATAATATTAAGAAA
Core staple54 AGCTATAATGAATTTAGAAAG
Core staple55 GAATTGAGTTAAGAGAACTGTAAAACTATGATAT
Core staple56 TTAATATTTTGTTAATAATCAAACCGAGCGCAAAG
Core staple57 CTGGTCAATTAGAGCTTTGCCATTATCAATCAATATATTAGGATTAGC
Core staple58 CGGGAGAAGCCTTTATTTCAAAACTCCAACAGGTCAGGATTA
Core staple59 ATACAGGTGCCTTGCAAGAACGACCAGG
Core staple60 ATATCAGAGAGATACCTTATTAGAATCGTGATAAA
Core staple61 TAGGAATCATTAACGAGCG
Core staple62

TCAACCGAGACTTCTATTGACGGAAATTTAGCAAATAAGACTACCCACAA
Core staple63 GCTTTAAATGTTTAGTTTTCAGTTTGCC
Core staple64 CGGCCTTGCTTAACATTTCTAAAACGGGAGGTTT
Core staple65 ACGGCTACATAAAGGCTAGCATTCTTAATTCCATGTACCGTAAC
Core staple66
TGAGAAAATTTTTAACAAAAGGGCGACAAAAGAAAGAAACGCCAATGAA
Core staple67 TCAAGAAACAAAATCCACACCACTATGG
Core staple68 ATCAAAACGCCGCCCCTCTACGTTAATATAATGCAAGTAAGATTTGCC
Core staple69 ATGCCTGCTGATCGGCAAAA
Core staple70 CTGTTTGGATAGGGCGGCTTAGACGCTG
Core staple71 CGCGAGCTCGTCTTTATTGCGA
Core staple72 AATTACTATAAGTCCTGAA
Core staple73 TTGCACGTAATCCTCATCACGTAGTAAT
Core staple74 GGAACCCTCGAGAGGCTTAGCGTCAATCCCCAGTCAGAAGAGAT
Core staple75 GACAAAGTAACGAAGGCACCAACCAATCGTCCATATA
Core staple76 TTGCTTTAGGAGGCTAAAGAAAAATTAT
Core staple77 AGTTGAAAGGAGCATTTAAAAGAAACCATGGATTACATA
Core staple78 CAGCTACGCGAACAAAGTTTGAGGGTAATATAAGAATACGTGGCACA
Core staple79 GAAGGAAAGGGAGAACCAAGTTAACAAA
Core staple80 ACAGTAGTTGGGAACTATTATCTCAAAT
Core staple81 GCCCGAATTCTAGCATGAACGCAATAGGAACG
Core staple82 GTGTGAAATTATGTTCAGCTAA
Core staple83 ACAGTTAGCCACCCATAGCCCTGCCGTCTCTGAAACCCTGCCGGGT
Core staple84 TTATAAATCAACTATATCATAGCGATTCCAAGAA
Core staple85 TTCACAAAGCGCAGCAACTTTGAATAAGGCTTGCCATCTTGAAGTAAC
Core staple86 GGGACGACGTTCATCAGTTGAGATTATTACACCCTCAAATCCTCAT
Core staple87 AATTGCGTAAATCAGTAGCACATGAAACCATCGATTTTCGGTTGGGTAAT
Core staple88 CAACGCGCAGTATGTATTCATTAGAGGAA
Core staple89 TGAGGAAGTTTCCAACGCATATTTTAAACATTTCG
Core staple90 CCTAGATGGGCGCATCGTACCGGGTAC
Core staple91 GCCTTAAATCAAGATTAGT
Core staple92 GAGTAACAACGGAAGCAAAAAGGTATTCTTA
Core staple93 GTGGCTGGCCCTCTTTTCCGCGCGGCAGTCGGGCTCACACAAACGG
Core staple94 CCTGATTACTCGTAAAAAACGCATTCTGGCCAACACTAAAACTCAATAT
Core staple95 GAGGATCCACAGACGGGCAA
Core staple96 ACATATATTCAACCGCTTCAA
Core staple97 AGCAACAAAGTCAGAGGGTAATTGTAGGCAGTGAGAGTGGTAGCT
Core staple98 GTCCAAATCAGGACAGATGAACGACTGACCCTATTATGAGAGGGTT
Core staple99 CGGGCACCGCTTCTGGTGCGCGATTAA
Core staple100 GGTAAAATACGTAACAATGACACGGTGTATTTAGT
Core staple101 TTTAATTTAAAGGACCAGACGTTAGTAATTTTCATTTGGGG
Core staple102 CCTCATTTTCAGGCAACATGACCGATATGAATTT
Core staple103 AGCATAAAGCTAGCGGATGGCTTA
Core staple104 TCCTGAATAAACCTACTACTTCTCACCGAGGAGT

Core staple105 CATAGCAGCACAGCAAAAAAGCCATATAAAGGAGAATT
Core staple106 GGCCTCTTGCAGAACTCAAA
Core staple107 TGCTATTTGTATCTTTAGGAATTATGGCTA
Core staple108 CAGAACGCTTAATGTTGAATAACAATAA
Core staple109 GATAGCCGAGATAGGCCATTGCTTTGATCAAATTATATTTTAGACA
Core staple110TAAGCAGATAGCGATTGTAGTAATGTGTAGGTATTTAAATTATGGTT
Core staple111ATAGAAAATTCAGCAATGCCT
Core staple112GAAAGACAGCATCGGACGTCACCACTCAATTCTACTATTCGTCA
Core staple113CCTTCCTCATTGCCAGGCATTTTGAGAA
Core staple114GTGCGTGCATCTGCCAGTTAGGTCGAC
Core staple115GAGATTCGCCATTCAGGCTCGCTATTA
Core staple116TGTCTTTCCTTAACAGCCA
Core staple117GCAGATTCAAAGATAACTAAAGCATCACCTATTGATTCCCAA
Core staple118GGGGTTTTGATGATGTACACTCATCTTTGAATCCGCCGCAGACGCTGGC
Core staple119AAATCACAATTCGAGATTGAGGGAGGGAACATAAAACCCAAACCCAATA
Core staple120 GTGTCGAACCCCCAGTGTATCACCGTCAGCTCAGTGAGGCTGGCCGTATA
Core staple121 CCGCGGAATAAACCAAAATAGCGTTTACCAAACCAGATCAGAGCCA
Core staple122 CAAATGGTTGCTAAACAACACAGTGCCATTAGATATATGCAAGCCACCAC
Core staple123 AACAGTTAATGCCCATGAAACATAAGGATAAATT
Core staple124 TTGACCACGCTGAGATTAACACCGCCTGAGCTTGAATCGCCCTTAAACG
Core staple125 AAGAGTACTGACGAAGATGGTTGGGAA
Core staple126 AAGAGTCGGGCGATATTAATTAACCTTGCTTCTGTTTTCATTCGGCGA
Core staple127 AGGACGTTTAATTTTCTCTGATTTGATG
Core staple128 CAGTAGCGTAGCGCGACTGGATTTGCAA
Core staple129 CGATTTTGAGAAAAAGATAAACACGGCCAAACCA
Core staple130 TGCAGAAAATAGCAGCCTTAAACAACGTTATCCGAAA
Core staple131 AGCATGTAGAAATATCCCA
Core staple132 ATATTTTTGAGAGGAAGG
Core staple133 GTTGTTAGAGCAGGCCAAGCGGACTGGGCGCCAGGG
Core staple134 CCAGGTAGATTTTAAGAACTGGCGTGAATTAAGCCAGTGGTAATAA
Core staple135 TAGATAACCCTCAAATCGCCATTAAAAAAAAGGGACTCATGGAATACA
Core staple136 AGAGCAACTGTTGGGAAGGGCGATCGG
Core staple137 ATTAATAGCGTCATAGATTGCTCGTCATC
Core staple138 AATCGGTTGTACCAAAAACATTTTTGATAAGAGGTCATTTTT
Core staple139 GTTTTGTGAAAAGGTGGCAAGACAGCCCTCATA
Core staple140 CCATCAAAAAAAACAAGACCAACATGTAATTAGCGCTA
Core staple141 AATTTGCTAATATGTGTAATGGATTAGAGCGGAG
Core staple142 TCATCAACATTGGGGTGAAGAGAAACGCTCA
Core staple143 TGGGATTTCAATAATGTAGCTGATAGCAAGCCCAA

Core staple144 TATATTGTCACCACATTACCTATACAAAAAGTAAACAGGGAAGCG
Core staple145 GCAAGTGTAGTTTCAATTCAGATGGCGCCCAATA
Core staple146 CGGATTGACCGTCTGTTTC
Core staple147 AATACATAGGTAAAAAATATCGCGTTTTCATCAATAGCATGTTAAATCA
Core staple148 CAGGTCAGACGTTCCAGGGGCTTGGAAACAC
Core staple149 TCAATTGCGTAGTAACAAGGCGAATGATGAACCTTTTTAGT
Core staple150 ACCGGAAGCACGCAAGGATA
Core staple151 TTAGTCTGAAAGCGTCCAGAACTTGCCTTAAAAGAGCCAGAAGCTA
Core staple152 TATGCGTATTAGCACGGTTTGCGTATCATTGCGCTACGAGCCCCGTCGG
Core staple153 TTAATGCAAGATTAAAAGGTGAATTATCATTTAA
Core staple154 TCGCCATACCGTCACGGATTGATAAATA
Core staple155 AACGCCAACCATCAAGCATTGACAGGATTCAATAGACCTTTTCGAGAA
Core staple156 CTCGAATTGTGTCGTGCCAG
Core staple157 ACGGCCAGATGTGGACTCCA
Core staple158 AACTGAACACCCTGCAGTAATCCAGGCTATCAGGTGTAGCCAGCTT
Core staple159 TATTCTAAGAACAATTTTA
Core staple160 GGATAAGGGAATAGGCGATTAGAAAGAG
Core staple161 GAATAATTTTCAGATTAAGGTTGGGCCAATCGCTTCTGATGATAA
Core staple162 ATAGCAATAGCTATAGAAGGAGAAAAGCAAAAGGG
Core staple163 TATGACCCTGTAATACTTTTGGAGAGTACCTTTAATTGCTCC
Core staple164 CTAAAAGCAATACTTCAACAGGTTAAATCCCGTCAA
Core staple165 TACCAGCGGAATAAAGTTACCCTTACCGAAGCCCTTTTTAAGAAAAG
Core staple166 ATTCAACAAACGAAACCACCACTTGATA
Core staple167 TTAGAATCAGCAGGTTTGGGTTAGGAAGGCTTAT
Core staple168 AGTGCCCTCATGGCTATTTACCGATTGGCCCAGAGCTCAGCCGCCAC
Core staple169 TGGTTTTTGAGAGAATTTCATCAAGACAAAGAACGTAACCTCTTGAGT
Core staple170 CGCCACCTTTCATTCTGAAATGGATTGATGCCACTCA
Core staple171 ATTCTCCGTGGGAAATTCCACCAGACGAAGTATCA
Core staple172 AGAGGGGTTTGCCCCGAATTACAGAGACTTGAATTT
Core staple173 ACGTGGTATTTTTTGTACGTGAAACCAGTTTGGAAC
Core staple174 AACTTTTAGCCCCCTAGGTCTGGAGGCATGATACAT
Core staple175 CAAAGGGCGAATCCTTGAATCAATCCGCACTCAT
Core staple176 CGGATTCAGACTCCAACGAGGGACCTGC
Core staple177 ATCTTTTCCGGAACTATCATAATACCAC
Core staple178 CTGTAGCATTCCCTCAGCACTCCAAAAAAAAGGTTTTTCATCTAAA
Core staple179 ATTTTTGAGCAAAGCCGACTTTCAAGTTTG
Core staple180 ACACCACGCCAAAGGAACCCT
Core staple181 CATTAGACGGTACCGACTAAAGTGTCACATT
Core staple182 AGTCACGAGCGGAACCCTAA

Core staple183 ATCACTCCAGCCAGCTTTCCAGGGTTT
Core staple184 TGATTGCCCTTTAATGGATGCTGAAATCAATAAT
Core staple185 TATTATTCCTGCAAATTTATATAAAAGAATAAAG
Core staple186 CGCTTTCGGAGAGGAGCGTTAAATAAGACCTGTTTCGACAATTACAGAG
Core staple187 CAGAGTGTACAATGGAAACAAATAGAGCCGCGCCA
Core staple188 CCAGTACAAACTACAACGC
Core staple189 TCTTTCCGTAAGAAGATTATTCACGGTCACACGT
Core staple190 AACGCTACCAATATACAGATTTTAGCGGGATCCT
Core staple191 AGTTGCGGGTCAGTAGCCAGCAGCAAATCGAGTAGCTGGAAGCTCAGAAC
Core staple192 TCAGTGAGGCGAATAATTTTTGCGGAGGCGTTTT
Core staple193 AGCAAATATTTAAATTACAGGAACGAACAAGTTTATTTTGTCACAATCA
Core staple194 ACAACTTTGGGAGATTTGTATCACGCGAAATATAAGTTCAGAAC
Core staple195 TTGAGGACTAAAGACTCGCTGAATAATGCCCTGTTT
Core staple196 CATATATAAGATTCCCCAAAAGTAAACG
Core staple197 AGGGAAGAAGTAACCACGCGCAGGTACCTTTTACATCACAGAAACGATTA
Core staple198 AGTAAAACAGTTCGGTCTTTAGCTAACTAAAGCCTAAATGTGAGC
Core staple199 ACTGAGTATAGTAGCGCTTTTAGGAGCC
Core staple200 GGTGCCGGGAAAGCTGAATTAACAAACA
Core staple201 ATCCAAACCGACCGTGCCTAAATTCACCGCTTCC
Core staple202 TCCATGTACCGTTGGCAATTCTCATATT
SH_104 AAAAAAAAAAAAAAAAAAAAAAAAAGCATAAAGCTAGCGGATGGCTTAAACAACAACAACAACAACAACAAC
SH_151 AAAAAAAAAAAAAAAAAAAAAAAAACCGGAAGCACGCAAGGATAAACAACAACAACAACAACAACAAC
His-apt GGCTTCAGGTTGGTCTGGTTGGGTTTGGCTCCTGTGTACG

**Aptamers**	**Sequence(5‘-3’)**
Aptamer #1	GUAGCGGGAGAGGCCGCUCCGAAUGAAA
Aptamer #2	GUAGGGGGAGGGACCCCUACGAAUGAAA
Aptamer #3	GUAGCGGGAGUCGCCCCAACGAAUGAAA
**shRNA**	**Target Sequence(5‘-3’)**
shTGFBR2-1	GACCTCAAGAGCTCCAATATC
shTGFBR2-2	CGACATGATAGTCACTGACAA
shADAMTS3-1	GCAGACTAACTGTGCTTATGT
shADAMTS3-2	GCATGTGGAAGAATGCTAATC
