# Supplementary material for: ADAMTS3 Promotes Hepatocellular Carcinoma Cell Growth and Migration Through TGF-β:TGFBR2 Signaling
Source: Cells. 2026 Sep 30;15(19):1784. doi: 10.3390/cells15191784 (PMC13664179; doi:10.3390/cells15191784)
Supplement: Supplementary file 1 [file cells-15-01784-s001.zip › Figure S2.pdf]

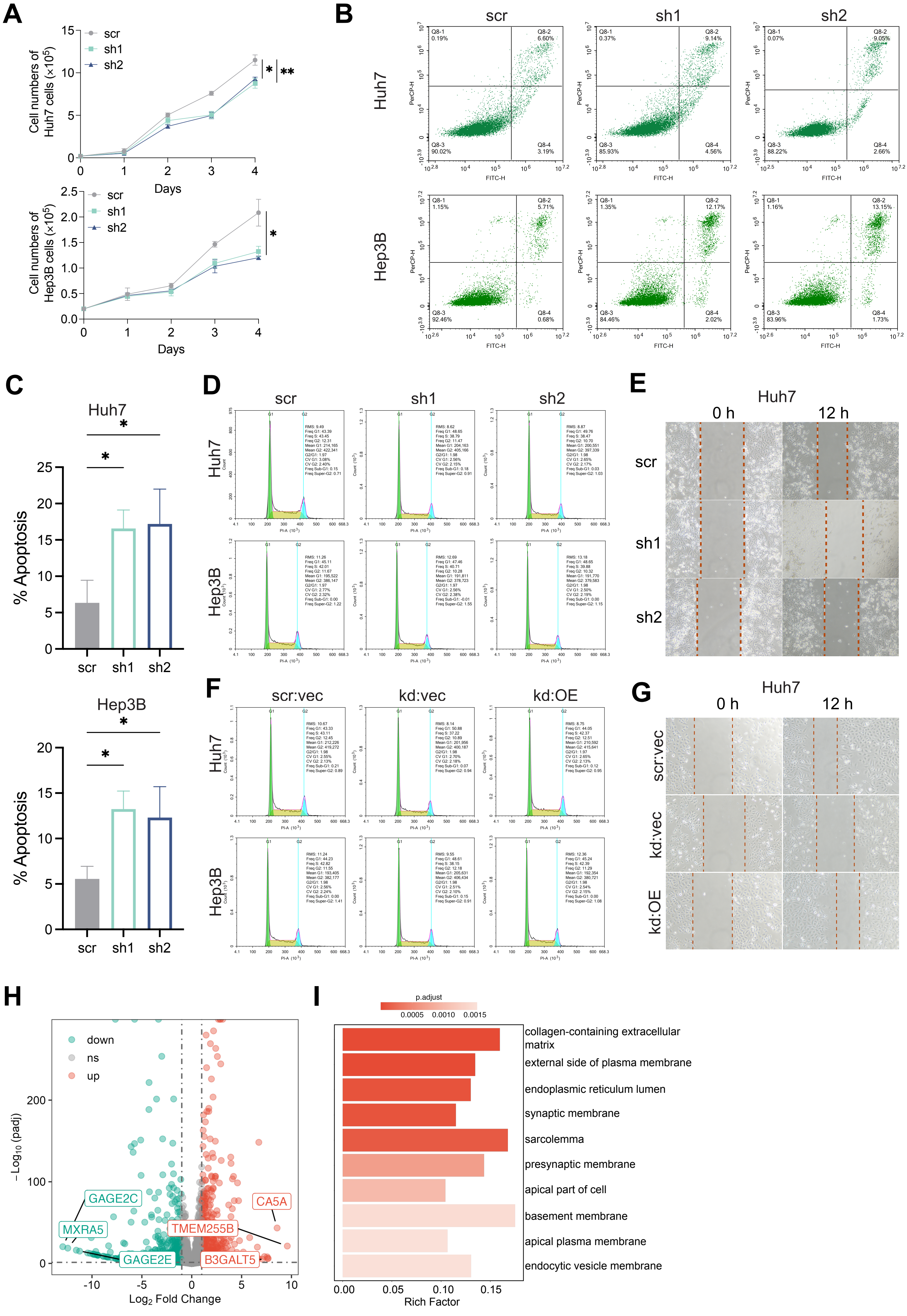
A
Cell numbers of Huh7 cells (×10^5)
scr
sh1
sh2
Days
Cell numbers of Hep3B cells (×10^5)
B
scr
sh1
sh2
Huh7
Hep3B
PerCP-H
FITC-H
C
Huh7
% Apoptosis
Hep3B
D
E
Huh7
0 h
12 h
F
scr:vec
kd:vec
kd:OE
G
H
down
ns
up
−Log10 (padj)
Log2 Fold Change
GAGE2C
MXRA5
GAGE2E
CA5A
TMEM255B
B3GALT5
I
p.adjust
collagen-containing extracellular matrix
external side of plasma membrane
endoplasmic reticulum lumen
synaptic membrane
sarcolemma
presynaptic membrane
apical part of cell
basement membrane
apical plasma membrane
endocytic vesicle membrane
Rich Factor
