# Supplementary material for: ADAMTS3 Promotes Hepatocellular Carcinoma Cell Growth and Migration Through TGF-β:TGFBR2 Signaling
Source: Cells. 2026 Sep 30;15(19):1784. doi: 10.3390/cells15191784 (PMC13664179; doi:10.3390/cells15191784)
Supplement: Supplementary file 1 [file cells-15-01784-s001.zip › Figure S1-S3 legneds.pdf]

Figure S1 related to Figure 2

A differential analysis comparing circulating tumor cells to HCC tissues.**B** ROC analysis of ADAMTS3 in distinguishing HCC from obesity and NAFL cases. **C** ADAMTS3 expression level in validation cohort.

Figure S2 related to Figure 3

A Growth curve of Huh7 and Hep3B based on cell counting. Cells were counted every 24 h after seeding. **B** Representative Annexin V/PI staining profiles showing apoptotic cell populations following ADAMTS3 knockdown. **C** Quantification of overall apoptotic rate following ADAMTS3 knockdown. **D** Representative flow cytometry profiles showing cell cycle distribution of Huh7 and Hep3B cells following ADAMTS3 knockdown. **E** Representative images of wound closure assays using Huh7 cells with or without ADAMTS3 knockdown. **F** Representative flow cytometry profiles showing cell cycle distribution of Huh7 and Hep3B cells in the ADAMTS3-knockdown background with or without ADAMTS3 re-expression.**G** Representative images of wound closure assays using ADAMTS3-knockdown Huh7 cells re-expression of ADAMTS3 or vector.**H** Volcano plot showing differentially expressed genes upon ADAMTS3 knockdown. **I** GO analysis on differentially expressed genes upon ADAMTS3 knockdown.

Figure S3 related to Figure 5

A. Binding responses of Aptamer #1 to ADAMTS3, ADAMTS2, and albumin. B. Numerical simulation illustrating the electromagnetic field confinement/enhancement between the AuNPs. C. TEM micrograph of two Au NPs self-assembled on the origami structure. D. Normalized intensity of ADAMTS3 at varying concentrations, measured on the DNA origami scaffold functionalized with Aptamer #1. E. Scatter plot of the normalized intensity, defined as the ratio of the protein signal at 1620 cm^{-1} to the internal DNA reference signal at 1095 cm^{-1}, versus the concentration of ADAMTS3.
